# Synthesis of Reabsorption-Suppressed Type-II/Type-I ZnSe/CdS/ZnS Core/Shell Quantum Dots and Their Application for Immunosorbent Assay

**DOI:** 10.1186/s11671-017-2135-4

**Published:** 2017-06-02

**Authors:** Sheng Wang, Jin Jie Li, Yanbing Lv, Ruili Wu, Ming Xing, Huaibin Shen, Hongzhe Wang, Lin Song Li, Xia Chen

**Affiliations:** 1 National & Local United Engineering Laboratory for Chinese Herbal Medicine Breeding and Cultivation, School of Life Sciences, Jilin University, Changchun City, 130021 People’s Republic of China; 2 Key Laboratory for Special Functional Materials, Henan University, Kaifeng, 475004 People’s Republic of China

**Keywords:** Type-II/type-I quantum dot, FLISA, Quantitative immunoassay

## Abstract

We report a phosphine-free one-pot method to synthesize ZnSe/CdS/ZnS core-shell quantum dots (QDs) with composite type-II/type-I structures and consequent reabsorption suppression properties. The as-synthesized QDs possess high efficient red emission (with quantum yield of 82%) and high optical stability. Compared to type-I QDs, the ZnSe/CdS/ZnS QDs show larger Stokes shift and lower reabsorption which can reduce the emission loss and improve the level of fluorescence output. The ZnSe/CdS/ZnS QDs are used as fluorescent labels to exploit their application in fluorescence-linked immunosorbent assay (FLISA) for the first time in the detection of C-reactive protein (CRP) with a limit of detection (LOD) of 0.85 ng/mL, which is more sensitive than that of CdSe/ZnS type-I QDs based FLISA (1.00 ng/mL). The results indicate that the ZnSe/CdS/ZnS type-II/type-I QDs may be good candidates for applications in biomedical information detection.

**Electronic supplementary material:**

The online version of this article (doi:10.1186/s11671-017-2135-4) contains supplementary material, which is available to authorized users.

## Background

Fluorescent core/shell semiconductor quantum dots (QDs) are characterized by the excellent optical properties such as broader emitting range, higher photoluminescence (PL) quantum yields (QYs), and higher optical and chemical stability than traditional organic dyes. These advantages open up opportunities for revolutionary advances in fluorescent labels for biomedical diagnostics, molecular imaging, and photoelectric field [1–7]. According to the band alignment between core and shell materials, the core/shell QDs can be classified as type-I, reverse type-I, and type-II structures. Type-I QDs characterized by the “nested” band alignment structure, which can confine both electrons and holes to the core region to enhance radiative recombination and physically separate the surface of the optical active core from its surrounding medium, and thus improve the PL intensity and optical stability [6–9]. Despite these favorable properties, a small Stokes shift (only a dozen of nanometers), referred to as the difference between the absorption and PL spectra, generates a serious reabsorption, leading to an overall emission loss and limiting their application in quantitative determination [10, 11]. In contrast, type-II QDs with staggered bandgap alignment promote spatial separation of electron and hole into different regions of the core/shell structure. The subsequent band edge e–h recombination transition energy is smaller than the bandgap of either of the constituent material components, leading to a significant red-shifted emission, which is unavailable with either monocomponent material. The oscillator strength of the first exciton absorption feature of type-II QDs dramatically diminishes compared to that of core QDs [12, 13]. The largely redshifted emission and the flatted first exciton absorption peak both lower the overlap of the absorption and emission spectra, which suppresses the reabsorption, and benefits the biological quantitative detection. The typical type-II ZnSe/CdS QDs have tunable emission from bluish violet to red range and suppressed reabsorption [13]. However, the electrons delocalized in CdS shell are vulnerable to trap from surface defects or surrounding medium, lead to low fluorescence quantum yield. A feasible solution is coating ZnSe/CdS QDs with ZnS outermost shell not only to passivate the surface for increasing the quantum yield and optical stability, but also to restrict the leak of toxic Cd element, reducing the biotoxicity. So far, the majority of researches have focused on type-I QDs, and only a few have been carried out on ZnSe/CdS/ZnS type-II/type-I QDs [12–15]. Moreover, all of the studies about the synthesis process of ZnSe/CdS/ZnS QDs used a two-step preparation by pre-purifying crude ZnSe core QDs and used toxic and expensive phosphines. In addition, none of them involved the application of ZnSe/CdS/ZnS QDs in biological detection.

Here, we report a phosphine-free one-pot method to synthesize high-quality red emission ZnSe/CdS/ZnS type-II/type-I core/shell QDs with the feature of reabsorption-suppression and the first using of the QDs to fabricate fluorescence-linked immunosorbent assay (FLISA). We used highly reactive and low toxic Se precursor (ODE-Se) and zinc oleate to synthesize high-quality ZnSe core QDs, and then achieved multishell growth without purification of core quantum dots. This shows great promise for large-scale synthesis of core/shell quantum dots. The quantum yield of as-prepared red emitting ZnSe/CdS/ZnS type-II/type-I QDs can reach as high as 82% with lower toxic cadmium content which is particularly important to reduce biotoxicity in biomedical field. Moreover, the QDs have large Stokes shift as well as flatted first absorption peak, which lead to low overlap of PL and absorption spectra and suppressed reabsorption effect.

C-reactive protein (CRP), as an acute phase protein from liver cells, has been regarded as an early indicator of infection and autoimmune disorders. Such diseases often commence at very low CRP levels. Therefore, the sensitive quantitative immunoassay analysis of CRP levels in biological samples is of critical importance for diagnosis and monitoring the evolution of diseases [16]. Compared with traditional enzyme-linked immunosorbent assay (ELISA), FLISA is time-saving without enzymatic reaction and less sensitive to environmental conditions deriving from the optical quality of fluorescent QDs [17]. Hence, FLISA has become a new research hotspot of quantitative immunoassay [2, 18–21]. Here, we firstly demonstrated FLISA quantitative immunoassay using water-soluble ZnSe/CdS/ZnS type-II/type-I core/shell QDs as fluorescent probe. The limit of detection (LOD) for quantitative detection of CRP protein reached 0.85 ng/mL which was 15% more sensitive than that of the CdSe/ZnS type-I QDs based FLISA in control experiments. The high QYs, excellent optical stability, and low reabsorption effect may promote the application of ZnSe/CdS/ZnS type-II/type-I QDs in biomedicine and photoelectric field.

## Methods

### Chemicals

Cadmium oxide (CdO, 99.99%), zinc oxide (ZnO, 99.9%, powder), selenium (Se, 99.9%, powder), 1-octadecene (ODE, 90%), 1-octanethiol (OT, 98%), oleic acid (OA, 90%) Poly(maleic anhydride-alt-1-octadecene) (PMAO), and 2-(N-morpholino) ethanesulfonic acid (MES) were purchased from Aldrich. Paraffin oil (analytical grade), acetone (analytical grade), hexanes (analytical grade), and methanol (analytical grade) were obtained from Beijing Chemical Reagent Co., Ltd, China. NaOH, HCl, Na_2_CO_3_, NaHCO_3_, KH_2_PO_4_, Na_2_HPO_4_, H_3_BO_3_, Na_2_B_4_O_7_ · 10H_2_O, and Tween-20 were purchased from Shanghai Sangon Co., Ltd, China. Bovine serum albumin (BSA) and calf serum were purchased from Sigma. 1-ethyl-3-(3-(dimethylamino) propyl) carbodiimide (EDC), N-Hydroxysulfosuccinimide (sulfo-NHS) and the microplates were purchased from Thermo Fisher Scientific (USA). Mouse anti C-reaction protein monoclonal antibody and CRP antigen were obtained from Abcam (USA). All chemicals and solvents were used as received without further purification.

### Stock Solution for the Se Precursor (0.1 M)

Se (6 mmol) and ODE (60 mL) were loaded in a 100 mL three-necked flask, and then heated to 220 °C for 180 min under nitrogen to obtain yellow clear solution.

### Stock Solution for Zn Precursor (0.4 M) and Cd Precursor (0.2 M)

ZnO (30 mmol), oleic acid (30 mL), and 45 mL ODE were loaded in a 100 mL three-necked flask and heated to 310 °C under nitrogen to obtain a clear solution. The resulting solution was allowed to cool down to 140 °C for injection. The preparation process for Cd precursor was same to Zn precursor except the concentration was adjusted to 0.2 M and the reaction temperature was set to 240 °C.

### Typical Synthesis of ZnSe/CdS/ZnS Type-II/Type-I QDs

As a typical synthetic procedure, 4 mL Se precursor and ODE (15 mL) were placed in a 100-mL round flask. The mixture was heated to 310 °C. At this temperature, 2 mL of Zn precursor was quickly injected into the reaction flask. Aliquots were extracted at different time intervals to monitor the evolution of PL position which coordinates with the particle size of QDs. When the core nanocrystals reached the desired dimension, the reaction temperature was reduced to 230 °C for CdS shell growth. Without any purification steps, as the mixture of Cd precursor and 1-octanethiol (the molar radio of OT and cation is 1:1.2) started to be added dropwise with a syringe pump at a rate of 3 mL/h, while the temperature was elevated to 310 °C. The same process was applied to shell growth of ZnS. Aliquots of QDs were taken during the reaction to analyse the development of ZnSe/CdS/ZnS core/shell QDs. The as-prepared core/shell QDs were purified by adding acetone, and then redispersed in chloroform.

### Typical Synthesis of CdSe/ZnS Type-I QDs

CdSe/ZnS QDs were synthesized as described in our previous reports [7]. Subsequently, the process of phase transfer, QDs-antibody detection probes and the preparation of FLISA were same to them of ZnSe/CdS/ZnS QDs, which would be described below.

### Phase Transfer of ZnSe/CdS/ZnS QDs for Bioapplication

Poly(maleic anhydride-alt-1-octadecene) (PMAO)—an amphiphilic oligomer whose hydrophobic ends interleave with the organic coating on QDs and hydrophilic end groups are free to interact with the surrounding buffer—has been used to transfer hydrophobic QDs into pure water. The ZnSe/CdS/ZnS QDs and PMAO were mixed and dissolved in chloroform with sonication (molar ratio of QD/PMAO was 1:7). After that, chloroform was then removed by rotary evaporation at 45 °C. Then, an equal volume of 0.1 M NaHCO_3_ aqueous solution (pH = 8.5) was added to dissolve the QDs-PMAO. PMAO encapsulated ZnSe/CdS/ZnS type-II/type-I QDs show no fluorescence loss and have high stability in aqueous solution under a wide range of pH environments.

### Preparation of QDs-antibody Detection Probes

The procedure has been widely reported in previous literatures [1–3]. The QDs-PMAO were firstly conjugated with monoclonal CRP antibody through activation of these -COOH groups by EDC and sulfo-NHS. Next, a certain amount of monoclonal CRP antibodies were added into the QDs and dissolved in BS buffer and then were blocked by BSA. Finally, the product was washed by 5 mM BS buffer (pH = 8.0) under centrifugation. The QDs-mAb was stored in 50 μL BS buffer (5 mM, pH = 8.0).

### Preparation of Antibody-Coated Fluorescence Microplate

Primary antibody (the concentration of CRP monoclonal antibody was 1.8 mg/mL) was diluted with a carbonate-bicarbonate buffer (50 mM pH = 9.6, CB buffer) in each well of microplate. Subsequently, the microplate was covered with sealing film and incubated at 4 °C for 24 h. In order to remove extra coating antibody, the microplate was washed three times with a wash buffer (0.05% Tween-20 in 10 mM PBS, pH = 7.4). Then, excess binding sites were blocked with 0.5% (w/v) BSA in 10 mM PBS (pH = 7.4) for incubating overnight at 4 °C. This process ensured that all the available and remaining binding sides of the microplate wells were covered. The microplate was dried in a chamber with constant temperature and humidity for 24 h, then stored at 4 °C for future use.

### Quantitative Detection of CRP by Fluorescence-Linked Immunoassay

In each well of a 96-well microplate, contained coating antibody was added 100 μL of the standard antigen and diluted to a series of concentrations with the sample buffer. The plates were incubated at 37 °C for 30 min and then washed five times with a wash buffer. Next, 100 μL of the QDs-mAb probes were diluted with the probe buffer (10% calf serum (v/v) in 0.1 M PBS) into each well were incubated and washed same as the above-mentioned process.

### Characterization

Room temperature UV–vis absorption and PL spectra were measured with an Ocean Optics spectrophotometer (mode PC2000-ISA). PL quantum yields (QYs) were determined by comparison of the integrated fluorescence intensity of the QD samples in solution with that of standard of known QYs (Rhodamine 101 (R101) ethanol solution (0.01% HCl, QY = 100%) as the standard). Transmission electron microscopy (TEM) studies were performed using a JEOL JEM-2010 electron microscope operating at 200 kV. Phase determination of the products was carried out on an X-ray diffractometer (D8-ADVANCE) using Cu-Ka radiation (wavelength = 1.54 Å). The sizes of QDs and QD-antibody probe were recorded using dynamic light scattering (Nano-ZS 90, Malvern Instruments, UK).

## Results and Discussion

**Fig. 1:**
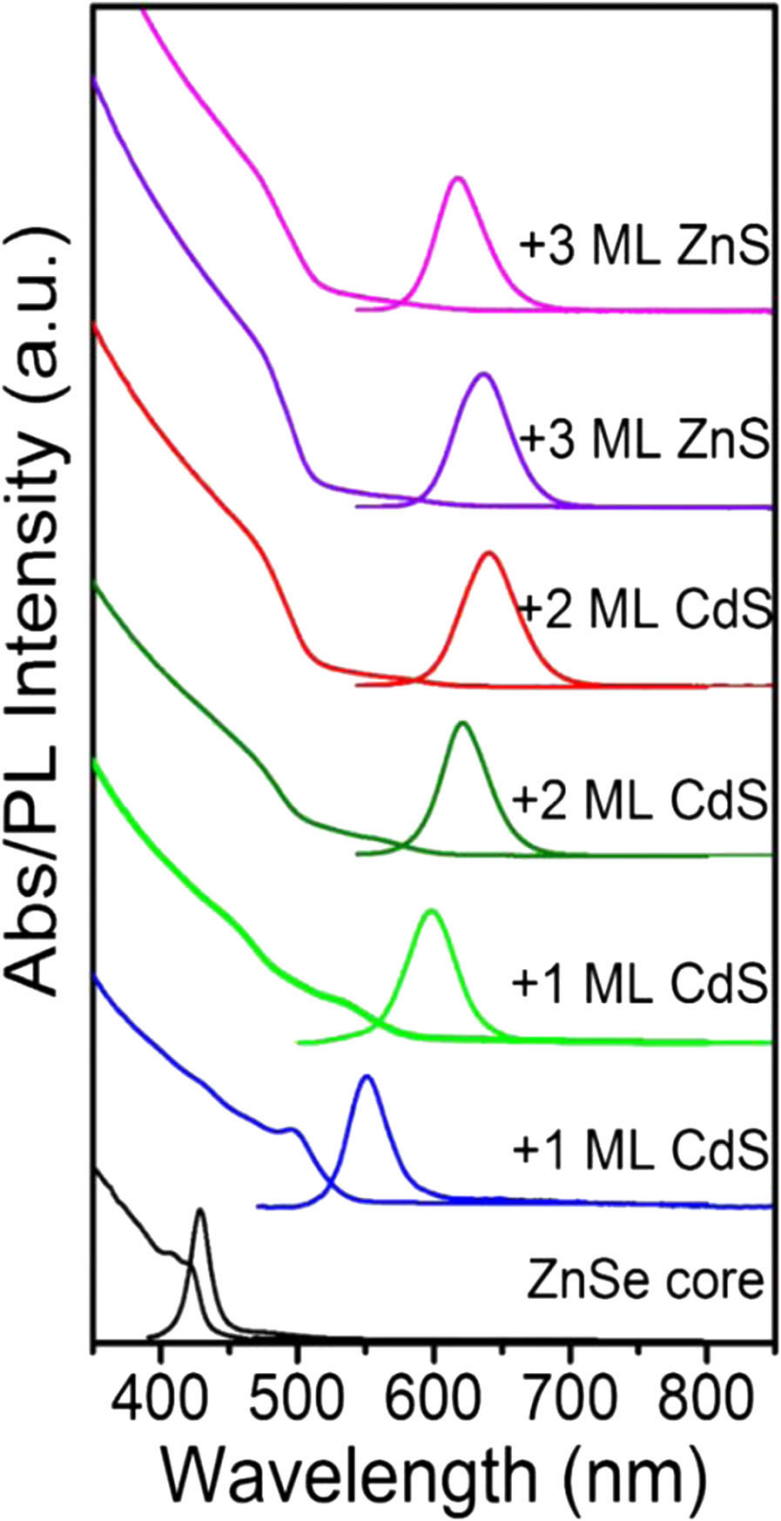
Evolution of the UV–vis absorption and PL spectra upon consecutive growth of ZnSe/CdS/ZnS core/shell QDs

The UV–vis absorption and PL spectra of shell growth process are shown in Fig. 1. The Stokes shift of ZnSe core is only 8 nm with the first absorption peak at 420 nm and emission peak at 428 nm, and emission full width at half maximum (FWHM) of 17 nm. However, when only one monolayer (ML) of CdS shell grew on ZnSe core, the Stokes shift significantly increased to 54 nm with the first absorption peak at 497 nm and emission peak at 551 nm, with emission full width at half maximum (FWHM) of 38 nm. Due to delocalization of the electron wavefunction, the PL emission of ZnSe/CdS QDs (629 nm) is redshifted with respect to ZnSe core QDs (428 nm), and the FWHM broadened to 52 nm with the deposition of CdS shell. The broadened PL FWHM originated from an enhanced Frölich-like exciton-phonon interaction [22, 23]. What is more, the oscillator strength of the first absorption peak rapidly weakened due to spatially indirect type-II transition from the valence band of the ZnSe to the conduction band of the CdS. The phenomenon is common in type-II QDs [24–26]. While the dramatic increase in absorption in blue spectral region (<500 nm) was assigned to the band gap of bulk CdS material (2.42 eV). In consequence, the red emission, the flatted first exciton absorption peak and the robust absorption in short wavelength region (<500 nm) of ZnSe/CdS type-II QDs resulted in large Stokes shift and suppressed reabsorption. With the sequential growth of ZnS shell, the PL was shifted to short wavelength and the FWHM was narrowed from 52 nm to 43 nm. This phenomenon was ascribed to the fact that the Zn-atoms diffuse into the Cd-rich regions to form gradient shell at high temperature, thus increasing the band-offset of the shell. The QYs could increase from 20 to 82% during the shell growth process of CdS and ZnS onto ZnSe cores.

**Scheme 1:**
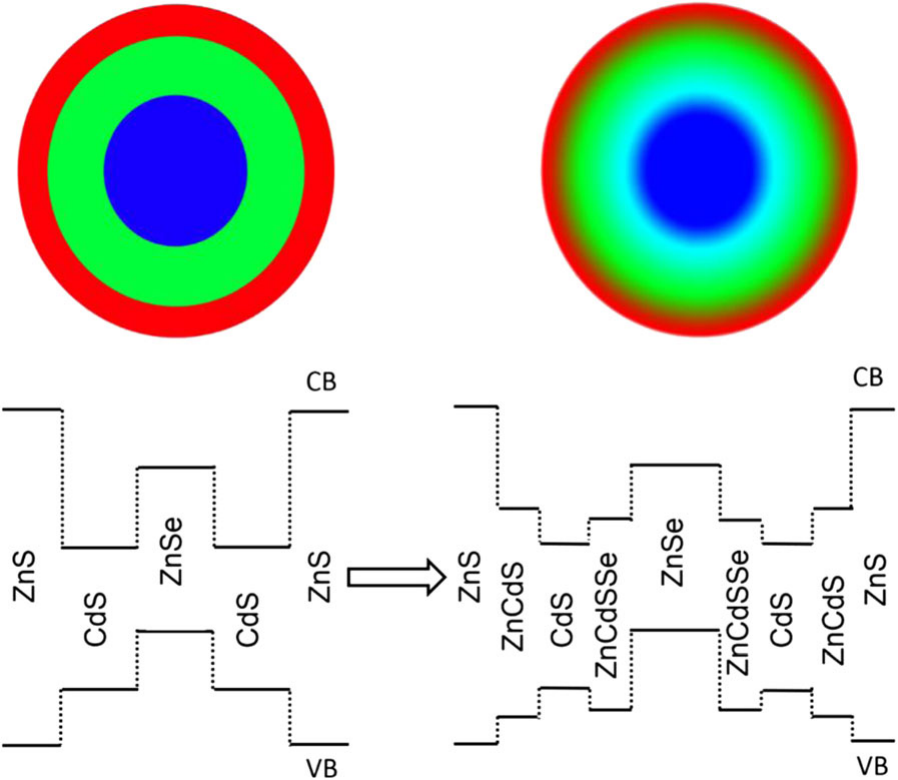
The schematic structure (*up*) and the band alignment (*bottom*) for ZnSe/CdS/ZnS type-II/type-I QDs based on the corresponding abrupt and alloyed interfaces, respectively

It was noteworthy that the excess of Zn-OA precursor relative to Se precursor in the core solution was necessary to obtain high-quality and monodisperse ZnSe core QDs. As a result, ZnCdSeS alloy shell would be inevitably formed during the addition of Cd-OT shell precursor in the initial stage since the high temperature (>200 °C) promoted the cation exchange and diffusion between Zn^2+^ and Cd^2+^, and the rich octanethiol in Cd-OT precursor could also react with the excess Zn-OA [7, 12, 27, 28]. The alloy shell can not only reduce the interfacial tension and defect to increase the QYs but also generate energy barrier for holes. The conduction band edge of ZnCdSeS alloy shell material was located between that of ZnSe and CdS, while the valence band edge was deeper than CdS. This formed a larger potential trough in the valence band edge as an additional blocking layer for the holes (Scheme 1) [12]. This energy band structure can further reduce the overlap of electrons and holes to decrease the strength of the first exciton absorption peak and suppress the reabsorption.

**Fig. 2:**
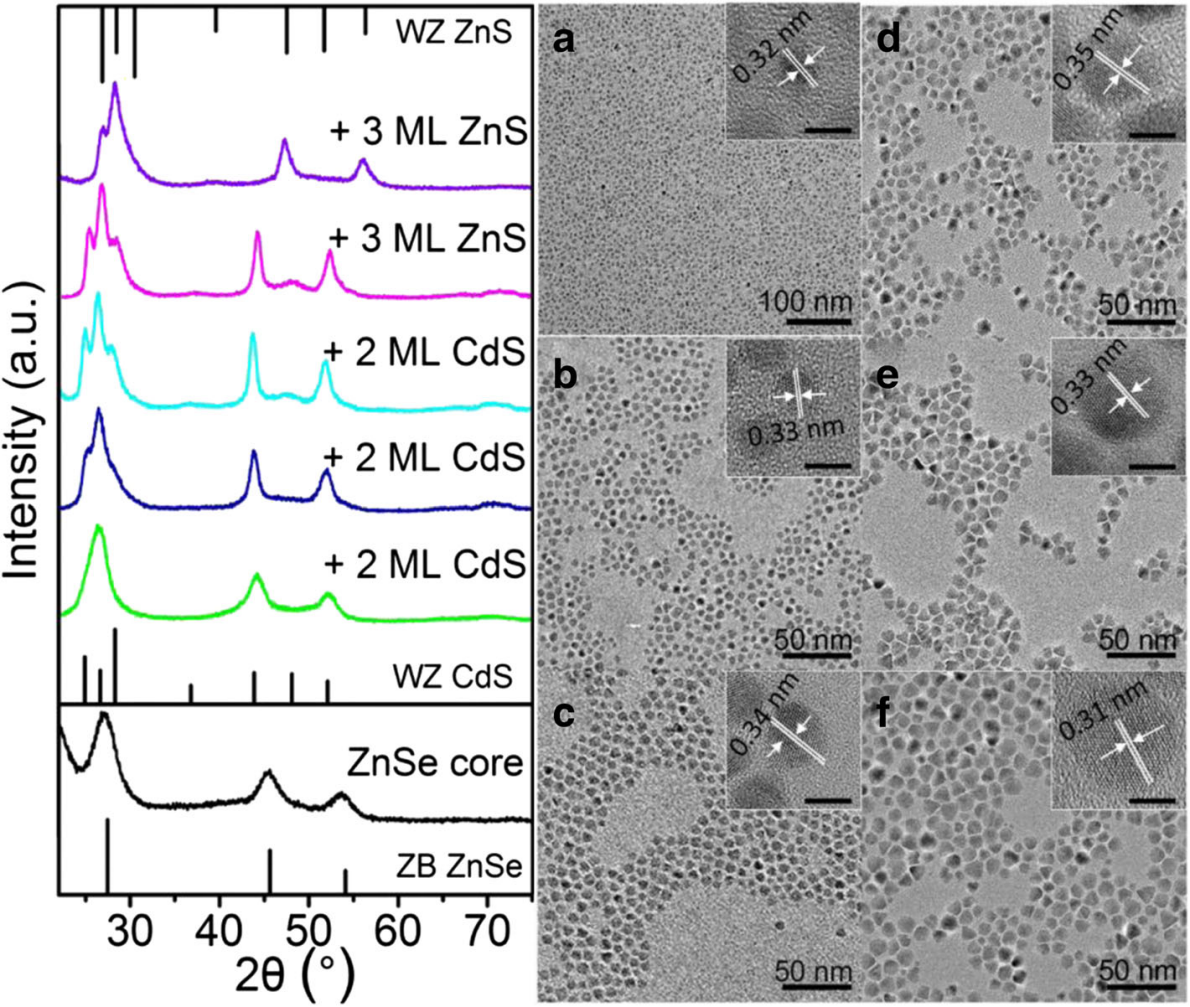
*Left*: XRD patterns of ZnSe/CdS/ZnS type-II/type-I QDs with different shell growth stages. The diffraction lines for zinc blende (ZB) ZnSe (bottom), WZ CdS (*middle*), and WZ ZnS (*top*) are indexed. *Right*: the corresponding TEM and HRTEM (*inset*, bar of 5 nm) images of the ZnSe core (**a**), ZnSe/CdS type-II QDs with 2 ML (**b**), 4 ML (**c**) and 6 ML (**d**) CdS shell, respectively, and ZnSe/CdS/ZnS type-II/type-I QDs with 3 ML (E) and 6 ML (F), respectively

The information about core/shell band structure and the evolution of PL and absorbance during the shell growth can be further verified by the comparison of XRD, TEM, and HRTEM of core and core/shell nanocrystals. The powder XRD patterns of the ZnSe core, ZnSe/CdS, and ZnSe/CdS/ZnS core/shell (the left picture of Fig. 2) show that the diffraction peaks become sharp and shift to positions corresponding to bulk wurtzite (WZ) CdS or ZnS crystal structures. This result is consistent with the predicted values for the larger volume of CdS or ZnS shell compared to ZnSe core in the final core/shell QDs and testifies the growth of multishells. Moreover, a transformation from zinc blende (ZB) type ZnSe cores to WZ-type core/shell has happened with CdS and ZnS coating. This phenomenon has been reported in CdSe/CdS core/shell QD systems [29, 30]. TEM images of the core QDs and several core/shell QDs are shown in Fig. 2a − 2 F. All the TEM images show nearly monodisperse spherical QDs with gradually increscent average diameters from original ZnSe core (3.90 nm) to ZnSe/6CdS type-II QDs (7.98 nm) and ZnSe/6CdS/6ZnS type-II/type-I QDs (11.92 nm). As shown in the HRTEM image of ZnSe core QDs (inset of Fig. 2a), the lattice spacing of the (111) planes is 0.32 nm, and the QDs possess good crystallinity and monodispersity. With the growth of shells, the lattice parameter showed corresponding change (0.35 nm for CdS and 0.31 nm for ZnS) in accordance with the XRD data. The results have clearly suggested the controllable growth of CdS and subsequent ZnS shell materials.

Meanwhile, to confirm the evolution of composition during the growth of multishell, the energy dispersive x-ray spectroscopy (EDS) analysis has been taken for different stages of the core/shell growth as shown in Additional file 1: Table S1. The EDS data show that the corresponding changes of content of Cd, Se, Zn, and S are in accordance with the shell growth stage. But it is worth noting that Cd/(Zn + Cd) molar ratio in the resulting ZnSe/CdS QDs is higher than the Cd/(Zn + Cd) feed ratio due to the cation exchange between Zn^2+^ and Cd^2+^ during the process of coating CdS shell onto ZnSe core at above 200 °C. By comparison with the issued literature about typical type-I CdSe/CdS/ZnS QDs (Cd molar ratio ~ 40%) [31], the type-II/type-I ZnSe/CdS/ZnS QDs contained far less Cd element (~13%).

A visual comparison of hydrophobic QDs in chloroform and PMAO-capped QDs in water under sun light and UV light is shown in Additional file 1: Figure S1 (A). It appears that both QD solutions are undisturbed and no aggregation of nanoparticles. Both QDs emitted the same red light when they were illuminated with a hand-held UV lamp (365 nm). Additional file 1: Figure S1 (B) shows the UV−visible absorption and PL spectra of QDs before and after phase transfer. Compared with the hydrophobic QDs in chloroform, the PL spectrum of PMAO-capped QDs has negligible change, indicating no obvious change in the particle size and PL properties. Additional file 1: Figure S1 (C) and (D) present the TEM images of the QDs before and after phase transfer which further determines the morphology and state of PMAO-capped QDs. It appears that PMAO-capped QDs are well isolated and rarely observed as aggregates.

In order to confirm the formation of PMAO encapsulated QDs during phase transfer process, FTIR spectroscopy was used to characterize the functional groups on the surface of QDs (shown in Additional file 1: Figure S2). The decrease of peak at 1777 cm^−1^ (compared PMAO with QDs-PMAO) and the increase of peak at 1715 cm^−1^ (compared the three samples) were attributed to the decomposition of anhydride and the formation of -COOH. The FTIR results indicated PMAO amphiphilic polymer was successfully coated on the surface of the ZnSe/CdS/ZnS QDs.

**Fig. 3:**
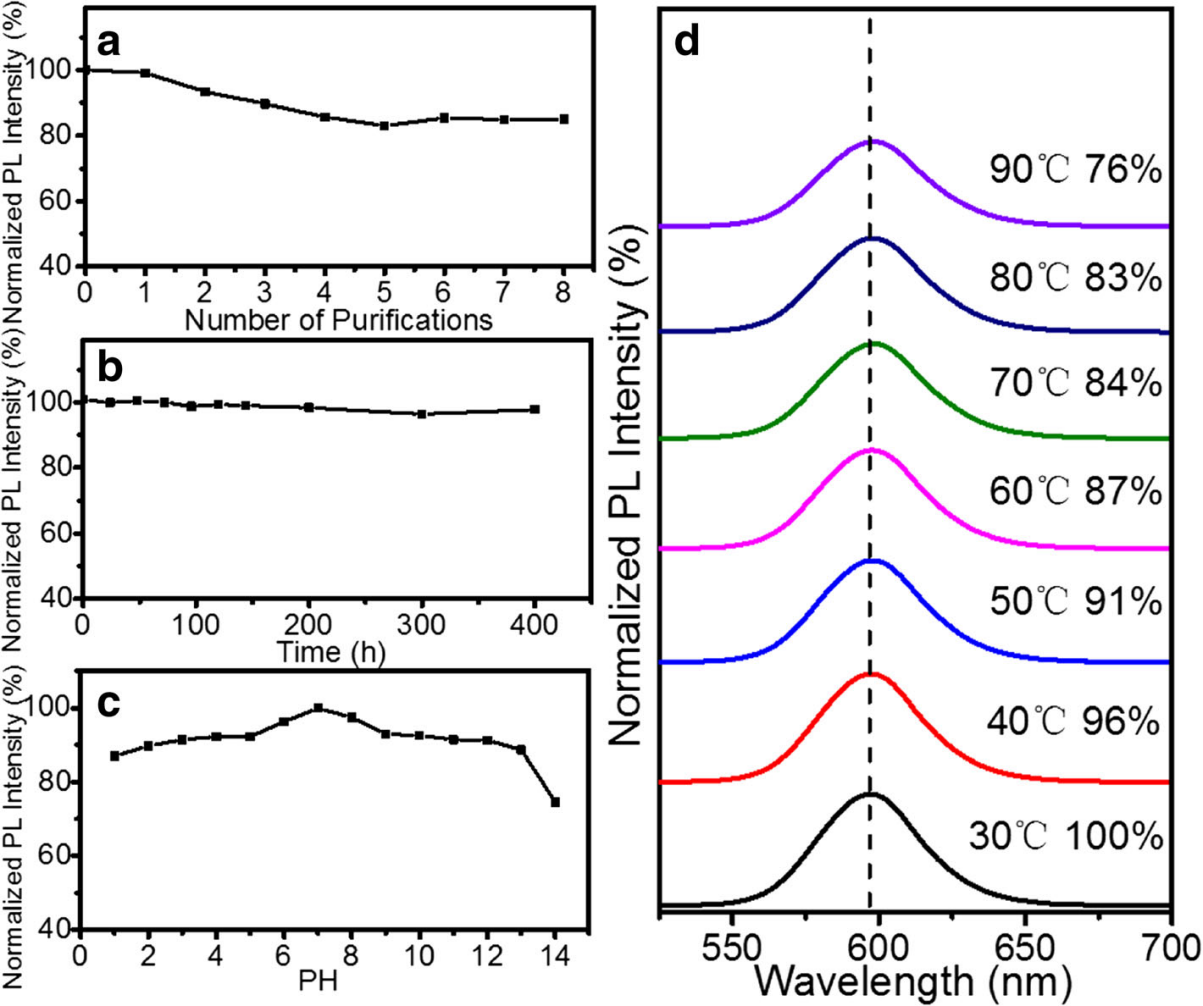
Stability test of hydrophobic QDs upon (**a**) repeated purification process steps; stability test of QDs-PMAO upon (**b**) BS buffer, (**c**) PH, and (**d**) temperature

The stability of as-prepared QDs is very important for the subsequent treatment. Figure 3a shows that the evolution of the relative PL stability of hydrophobic ZnSe/CdS/ZnS QDs upon purification steps. The PL intensity of ZnSe/CdS/ZnS core/shell QDs could maintain 85% upon many cycles of purification in hexanes. As shown in Fig. 3b, the colloidal stability of the QDs-PMAO in BS buffer (pH = 7.2) was estimated as a function of time at 25 °C. The PL intensity almost kept constant and the solution was clear even after 400 h. This indicates that the QDs-PMAO is stable in BS solution without any damage. Figure 3c shows the variation in PL intensity of QDs-PMAO which were immersed in acidic-to-basic pH (pH = 1-14, adjusted by HCl or NaOH) solution for 30 min. The PL intensity of hydrophilic QDs could retain over 85% except when the PH = 14. Figure 3d shows the effect of temperature parameter on the relative fluorescence intensity of QDs-PMAO. The fluorescence intensity gradually decreased with the temperature increase but still maintained 76% at 90 °C, while the PL peaks gradually shifted to longer wavelength due to the thermal expansion and electron–phonon coupling effect. All stability evaluation indicates that the ZnSe/CdS/ZnS type-II/type-I QDs and QDs-PMAO were very stable and thus suitable for biological applications.

**Fig. 4:**
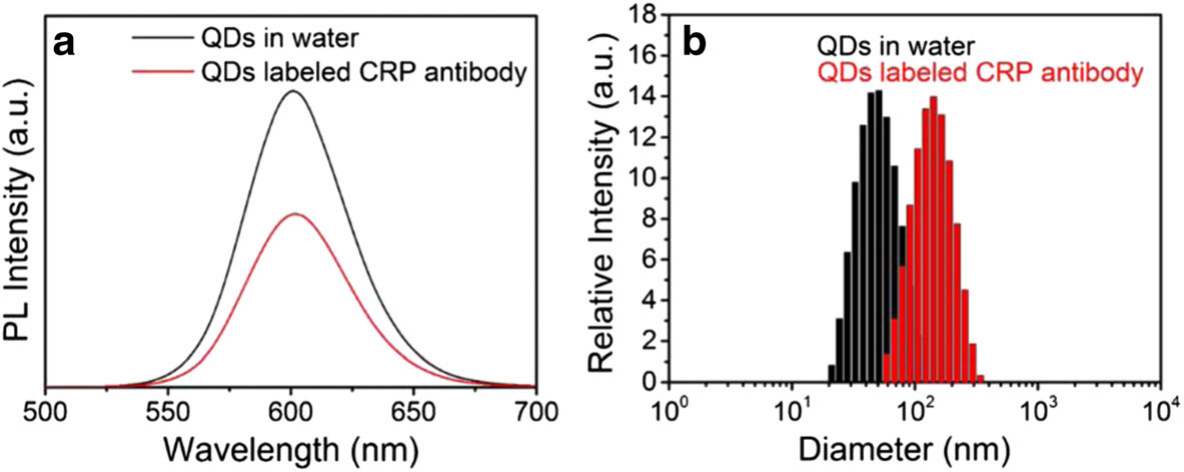
Fluorescence spectra (**a**) and dynamic light scattering (**b**) of the QDs-PMAO and QDs-mAb in buffer

CRP is an acute phase protein from liver cells, and the level of it is regarded as an early indicator of infection and autoimmune disorders. Here, the as-synthesized PMAO-capped ZnSe/CdS/ZnS QDs are coupled with CRP to demonstrate the possibility for application in quantitative immunoassay. Comparison diagram of the fluorescence spectra of aqueous ZnSe/CdS/ZnS QDs and the QDs-mAb are shown in Fig. 4a. Clearly, the PL peak shape of both samples are approximately identical except that the fluorescence intensity declines to 60% after coupling reaction due to inevitable sample loss during centrifuge separation process. It proves the excellent optical stability of ZnSe/CdS/ZnS type-II/type-I QDs even after the antibody protein coupling process.

To further investigate the effect of conjugation on the size of QDs, the aqueous QDs and QDs-mAb are characterized by dynamic light scattering (DLS). The DLS results (Fig. 4b) clearly show that both of the samples have narrow size distribution with good monodispersity and maintain a discrete form without aggregation, while the hydrodynamic size increases from 46 to 120 nm after coupling process. This demonstrates the success in conjugation with CRP antibodies.

**Fig. 5:**
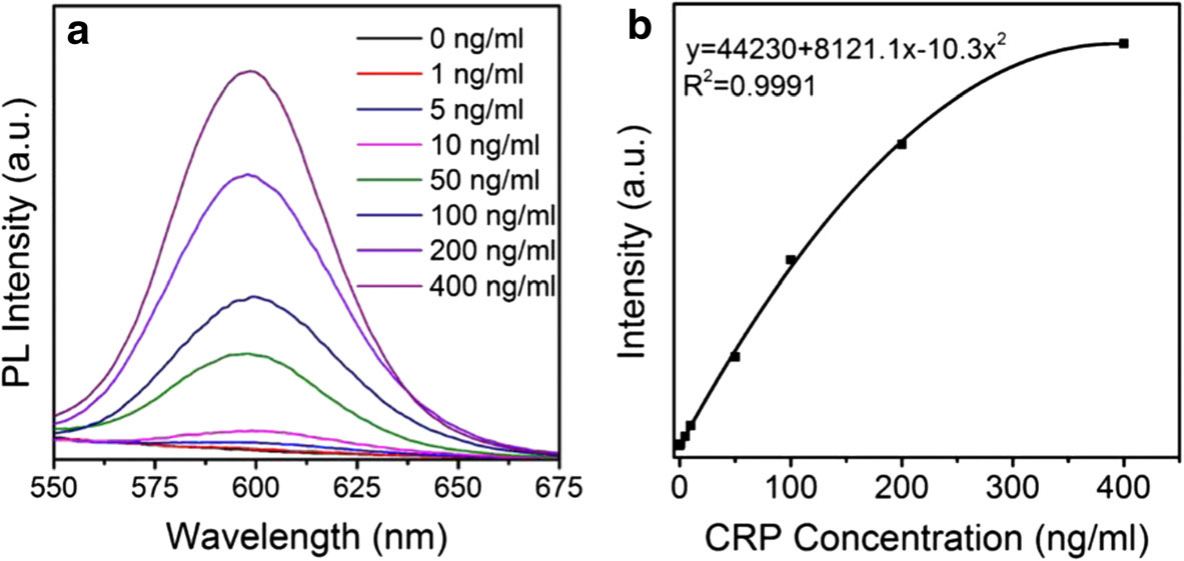
Photoluminescence spectra of FLISA for determination of different concentration of CRP antigen (**a**) and the standard curves (**b**)

Further, we used the ZnSe/CdS/ZnS type-II/type-I composite quantum dots instead of CdSe/ZnS type-I QDs as fluorescent probe to establish a FLISA for quantitative detection of CRP. The assemble process is shown in Additional file 1: Scheme S1. Figure 5a shows the relative fluorescence intensity of QDs fluorescence label for immunoassay in detection of various concentrations of CRP antigens (the standard CRP antigen is diluted to 0, 1, 5, 10, 50, 100, 200, 400 ng/mL). Obviously, the PL intensity gradually increases with the increase of concentration of CRP. Figure 5b shows that the correlation between the fluorescence intensity and the target CRP concentrations obey a quadratic regression curve equation of *y* = 44230 + 8121.1x-10.3x^2^ with a correlation coefficient of 0.9991, which is the closer to 1 the better. The working concentrations range from 0 to 400 ng/mL. The LOD is one of the key parameters for immunoassay for FLISA. By using the ZnSe/CdS/ZnS composite type-II/type-I QDs as fluorescent probe, the sensitivity of quantitative detection of CRP is 0.85 ng/mL, which is 15% more sensitive than that of FLISA based on CdSe/ZnS type-I QDs (1.00 ng/ml) (in Additional file 1: Figure S3).

Additionally, recovery experiments were used to evaluate the matrix effect of the FLISA with a series of known standard CRP antigens for analysis, and the final concentrations covered the low, medium, and high-risk levels. As shown in Table 1, all of the recovery rates are within the range of 83.61-105.9%. These results indicate that the FLISA based on ZnSe/CdS/ZnS type-II/type-I QDs with reabsorption suppression property has high accuracy and is of great advantages in quantitative immunoassay detection.

**Table 1:** Recovery tests for CRP determination

Sample	Experimental value (ng/mL)	Theoretical value (ng/mL)	Recovery (%)
P1	288.53	300	96.18
P2	148.73	150	99.15
P3	74.99	75	99.99
P4	26.32	25	105.90
P5	4.18	5	83.61

## Conclusions

We report a phosphine-free one-pot method to synthesize reabsorption-suppressed ZnSe/CdS/ZnS type-II/type-I core/shell QDs with large Stokes shift and flat first absorption peak. These characteristics reduce reabsorption and improve the level of fluorescence output. The as-synthesized QDs have high QY (82%) and high stability against various test conditions. Then, we first use ZnSe/CdS/ZnS QDs as fluorescence probe in FLISA for quantitative detection of CRP protein with high sensitivity (LOD of 0.85 ng/mL). It indicates that the reabsorption-suppressed ZnSe/CdS/ZnS type-II/type-I core/shell QDs have promising potential for application in biomedicine and photoelectric fields.

## Additional file

Additional file 1: Visual, PL, and TEM comparison of QDs before and after phase transfer. FTIR characterizations of hydrophobic QDs, PMAO and water-soluble QDs-PMAO. The EDS data of QDs with different injection volume of shell precursor. The scheme of assemble process of FLISA for quantitative detection of CRP. The PL spectra and standard curve of FLISA based on CdSe/ZnS QDs. (DOCX 1332 kb)

## Abbreviations

BSA: Bovine serum albumin
CRP: C-reactive protein
DLS: Dynamic light scattering
EDC: 1-ethyl-3-(3-(dimethylamino) propyl) carbodiimide
EDS: Energy dispersive x-ray spectroscopy
ELISA: Enzyme-linked immunosorbent assay
FLISA: Fluorescence-linked immunosorbent assay
FWHM: Full width at half maximum
LOD: Limit of detection
MES: 2-(N-morpholino) ethanesulfonic acid
OT: 1-octanethiol
PMAO: Poly(maleic anhydride-alt-1-octadecene)
QDs: Quantum dots
QYs: Quantum yields
sulfo-NHS: N-Hydroxysulfosuccinimide
