# Supplementary material for: Synthesis of Reabsorption-Suppressed Type-II/Type-I ZnSe/CdS/ZnS Core/Shell Quantum Dots and Their Application for Immunosorbent Assay
Source: Nanoscale Res Lett. 2017 Jun 2;12:380. doi: 10.1186/s11671-017-2135-4 (PMC5457375; doi:10.1186/s11671-017-2135-4)
Supplement: Additional file 1: — Visual, PL, and TEM comparison of QDs before and after phase transfer. FTIR characterizations of hydrophobic QDs, PMAO and water-soluble QDs-PMAO. The EDS data of QDs with different injection volume of shell precursor. The scheme of assemble process of FLISA for quantitative detection of CRP. The PL spectra and standard curve of FLISA based on CdSe/ZnS QDs. (DOCX 1332 kb) [file 11671_2017_2135_MOESM1_ESM.docx]

**Additional file**

Synthesis of reabsorption-suppressed type-II/type-I ZnSe/CdS/ZnS core/shell quantum dots and their application for immunosorbent assay

Sheng Wang,^a^ Jin Jie Li,^b^ Yanbing Lv,^b^ Ruili Wu,*^,b^ Ming Xing *^,a^, Huaibin Shen,^b^ Hongzhe Wang,^b^ Lin Song Li,^b^ and Xia Chen*^,a^

*^a .^*College of Life Science, Jilin University, Changchun City, 130021, P. R. China.

*^b.^* Key Laboratory for Special Functional Materials of Ministry of Education, Henan University, Kaifeng, 475004, P. R. China.


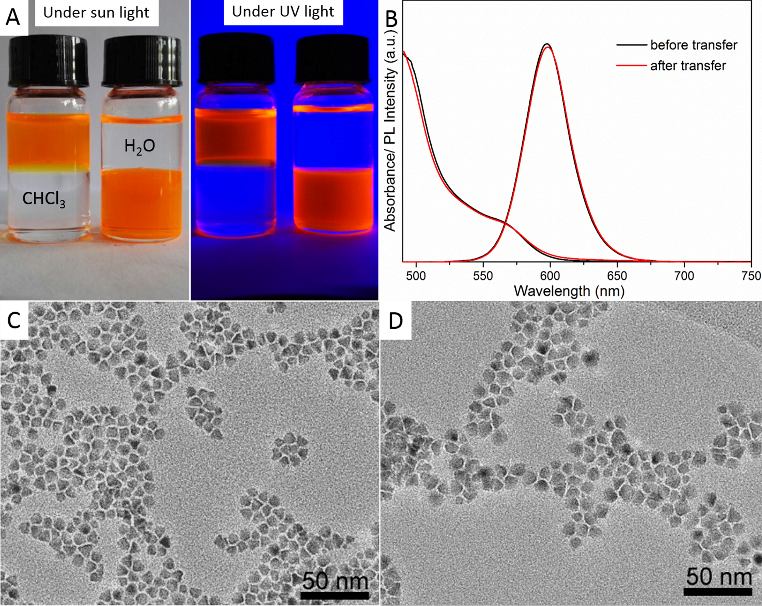


**Figure S1.** (A) a visual comparison of QDs before and after phase transfer in chloroform (top) and water (bottom) under sun light and UV light. The top solution is water, and the bottom solution is chloroform. (B) Comparison of the absorption (fixed the same OD value) and PL spectra of QDs before (black line) and after (red line) phase transfer. TEM images of QDs before (C) and after (D) phase transfer, respectively.


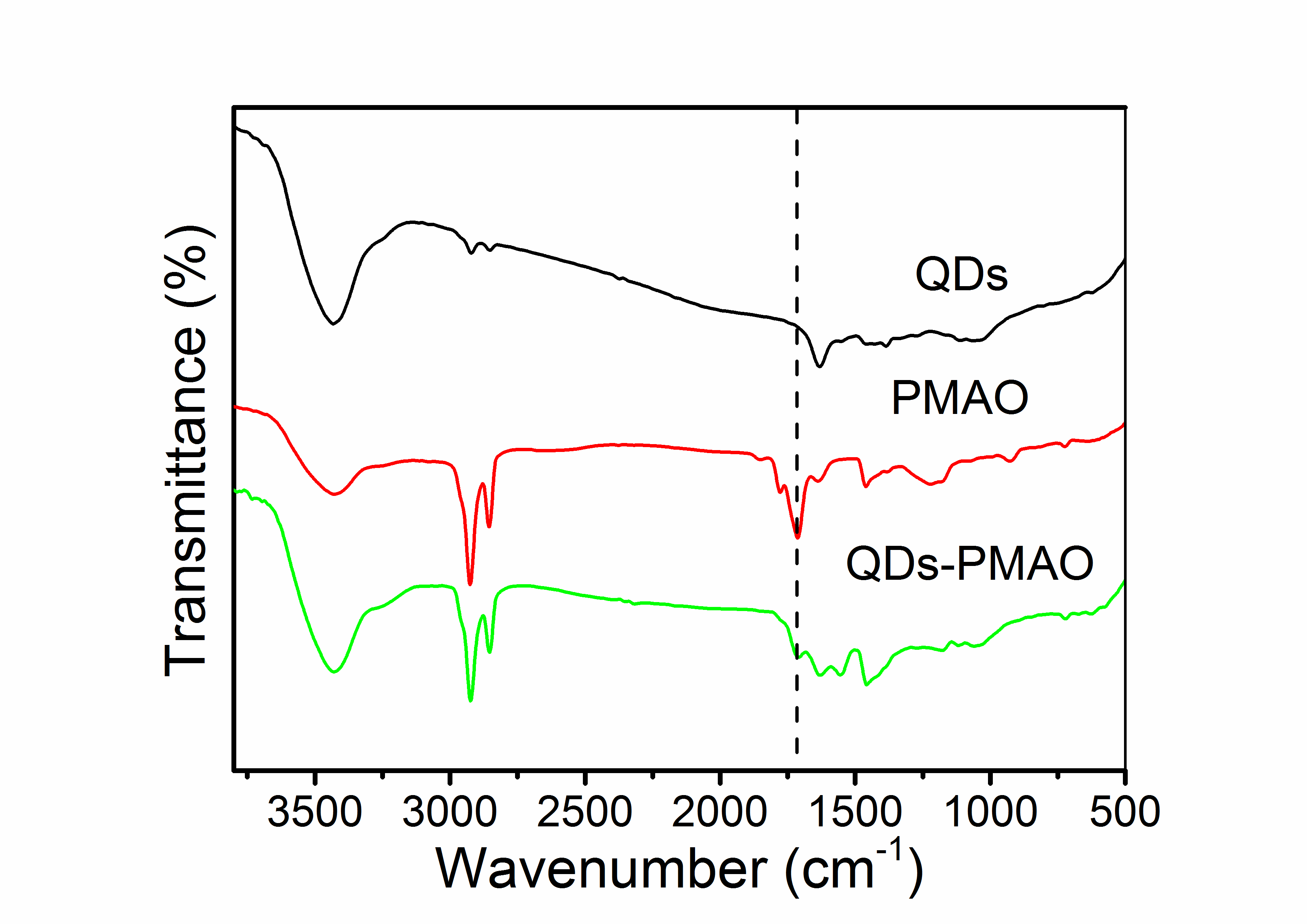


**Figure S2.** FTIR spectra of hydrophobic QDs (top), PMAO (middle), and water-soluble QDs-PMAO (bottom).

**Table S1.** EDS data of QDs with different shell thicknesses. Cd/Cd+Zn^ex^ refers to experimental result, and Cd/Cd+Zn^cal^ refers to calculative result according to the feed radio.

|  | Zn | Cd | Se | S | Cd/Cd+Zn ^ex^ | Cd/Cd+Zn ^cal^ |
| --- | --- | --- | --- | --- | --- | --- |
| ZnSe | 73.90 | -- | 26.10 | -- | -- | -- |
| ZnSe/1CdS | 20.32 | 22.42 | 44.79 | 12.47 | 52.5% | 50% |
| ZnSe/2CdS | 8.30 | 39.82 | 14.63 | 37.25 | 82.7% | 66.7% |
| ZnSe/4CdS | 4.98 | 41.49 | 9.29 | 44.24 | 89.3% | 71.5% |
| ZnSe/6CdS | 2.95 | 42.96 | 6.29 | 47.80 | 93.6% | 83.3% |
| ZnSe/6CdS/3ZnS | 8.62 | 33.90 | 2.98 | 47.72 | 79.7% | 59.8% |
| ZnSe/6CdS/6ZnS | 40.75 | 13.07 | 0.33 | 45.84 | 24.3% | 22.7% |


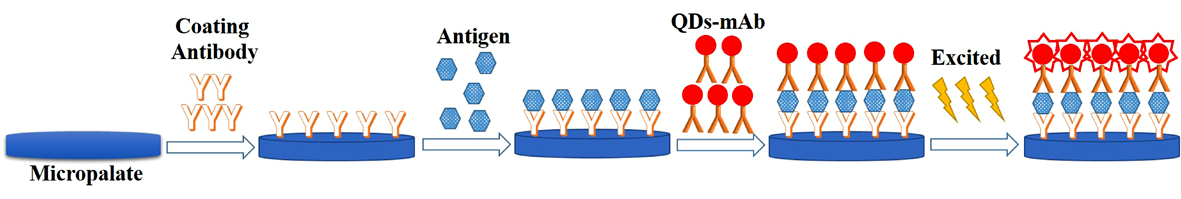


**Scheme S1.** The assembly process of FLISA for quantitative detection of CRP.


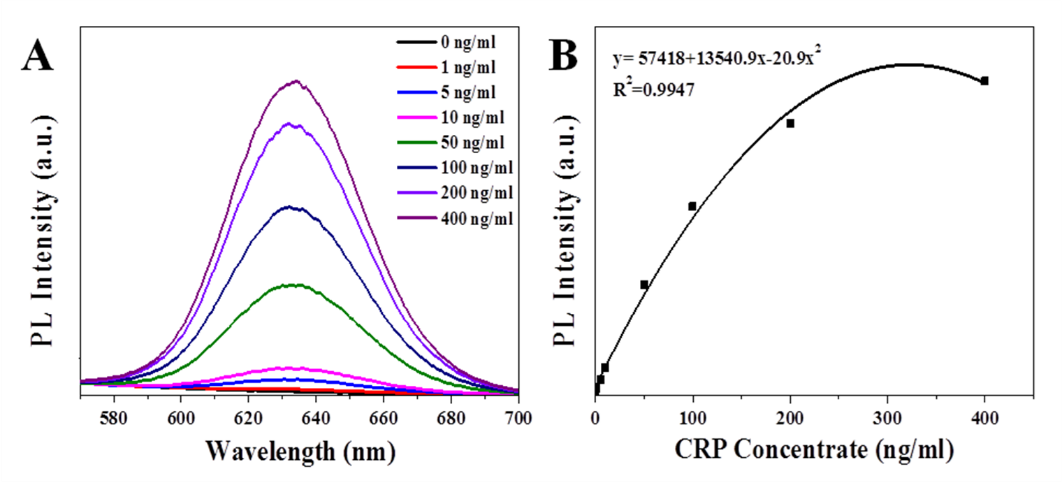


**Figure S3.** Photoluminescence spectra of FLISA based on CdSe/ZnS type-I QDs for determination of different concentrations of CRP antigen (A) and the standard curve (B).
